# Transportation Preferences of Patients Discharged from the Emergency Department in the Era of Ridesharing Apps

**DOI:** 10.5811/westjem.2019.5.42762

**Published:** 2019-07-02

**Authors:** Amar Tomar, Siddhi S. Ganesh, John R. Richards

**Affiliations:** University of California, Davis Medical Center, Department of Emergency Medicine, Sacramento, California

## Abstract

**Introduction:**

Patients discharged from the emergency department (ED) may encounter difficulty finding transportation home, increasing length of stay and ED crowding. We sought to determine the preferences of patients discharged from the ED with regard to their transportation home, and their awareness and past use of ridesharing services such as Lyft and Uber.

**Methods:**

We performed a prospective, survey-based study during a five-month period at a university-associated ED and Level I trauma center serving an urban area. Subjects were adult patients who were about to be discharged from the ED. We excluded patients requiring ambulance transport home.

**Results:**

Of 500 surveys distributed, 480 (96%) were completed. Average age was 47 ± 19 years, and 61% were female. There were 33,871 ED visits during the study period, and 67% were discharged home. The highest number of subjects arrived by ambulance (27%) followed by being dropped off (25%). Of the 408 (85%) subjects aware of ridesharing services, only eight (2%) came to the ED by this manner; however, 22 (5%) planned to use these services post-discharge. The survey also indicated that 377 (79%) owned smartphones, and 220 (46%) used ridesharing services. The most common plan to get home was with family/friend (35%), which was also the most preferred (29%). Regarding awareness and past use of ridesharing services, we were unable to detect any gender and/or racial differences from univariate analysis. However, we did detect age, education and income differences regarding awareness, but only age and education differences for past use. Logistic regression showed awareness and past use decreased with increasing patient age, but correlated positively with increasing education and income. Half the subjects felt their medical insurance should pay for their transportation, whereas roughly one-third felt ED staff should pay for it.

**Conclusion:**

Patients most commonly prefer to be driven home by a family member or friend after discharge from the ED. There is awareness of ridesharing services, but only 5% of patients planned to use these services post-discharge from the ED. Patients who are older, have limited income, and are less educated are less likely to be aware of or have previously used ridesharing services. ED staff may assist these patients by hailing ridesharing services for them at time of discharge.

## INTRODUCTION

Emergency department (ED) visits continue to grow steadily each year, with roughly 137 million per year based on the most recent data from the National Center for Health Statistics.^1^ ED crowding remains a serious problem despite progressive measures aimed at improving patient flow, especially with regard to inpatient admissions.^2^ One factor that has received less recognition as a potential variable for ED crowding is time spent arranging transportation home for patients discharged from the ED, which adds to overall length of stay.^3^ Discharged patients may experience difficulty finding family or friends to pick them up from the ED, may not be able to use public transportation, or may have physical or mental limitations on their ability to get home on their own. Patients who have driven themselves to the ED may have received sedating medications and be unsafe to drive. The onus of finding appropriate transportation home for discharged patients frequently falls on ED staff, who may be overextended during periods of crowding.

The advent and rapid growth of ridesharing services, such as Lyft and Uber, represents a potential solution for timely patient discharges from the ED.^4^ Once available only to those with smartphone or internet access, ridesharing services may now be arranged by telephone and arrive expeditiously and reliably via real-time global positioning system tracking via an application (app) or webpage. These services are usually paid for by patients but may be covered by medical insurance or individual EDs contracting with ridesharing companies. As this technology is relatively new, the impact of ridesharing services on patient transportation to and from the ED is not yet known.

To determine how much patients know about and use ridesharing services, we conducted a survey study in the ED. We also queried ED patient demographics, their preferences regarding transportation home, and their opinion of how it should be paid for. Our findings may be of interest to hospital and ED leadership, administration, and nursing. As ridesharing services expand and become more accessible to those without smartphone access, we believe ED patients may prefer this mode of transportation to and from the ED, especially if the hospital and/or their medical insurance cover its cost.

## METHODS

We performed a prospective, survey-based study during the five-month period September 1, 2017–January 31, 2018, at a university-associated ED and Level 1 trauma center serving an urban population of two million in central California. The study coincided with the advent of a hospital policy of arranging and paying for ridesharing service to certain patients discharged from the ED with financial or social hardships; the service was provided at the discretion and sanction of the ED charge nurse. This new policy was not publicized, did not apply to taxi services, and was granted on a case-by-case basis upon patient request. Subjects were a convenience sample of randomly-chosen adult patients 18 years of age or older, or adult parents of patients under 18, who were about to be discharged from the ED. The survey questions (Supplement 1) were only provided in the English language, requiring subjects to be able to read English or have their accompanying family and/or friends translate for them. Exclusion criteria were patients requiring ambulance transport home.

The survey was voluntary and anonymous, and potential subjects could decline participating in the study prior to receiving the survey instrument. Surveys were distributed to subjects in paper form and collected immediately after completion by collaborators from our Emergency Medicine Research Associate Program (EMRAP). The EMRAP collaborators clarified any questions subjects may have had regarding the survey and also checked the surveys for completeness. If any surveys were incomplete or had multiple responses checked, the EMRAP collaborators worked with the subject to resolve any discrepancies at the time of collection. The time period of distribution in the ED was from 5 AM to midnight, seven days a week. This study was approved by our institutional review board as an exemption, and patient consent was waived. We performed univariate and multiple logistic regression analyses using MedCalc™ version 18.11.3 (Ostend, Belgium). Statistical significance was assumed at P ≤ 0.05.

**Population Health Research Capsule**

What do we already know about this issue?

Patients discharged from the emergency department (ED) may encounter difficulty obtaining a ride home, especially the elderly and disabled.

What was the research question?

What are patients’ preferences regarding transportation home, and what is their awareness and use of ridesharing services?

What was the major finding of the study?

Transportation home with family or friend is preferred. Awareness and use of ridesharing services is limited.

How does this improve population health?

Ridesharing services are underutilized, and ED staff may arrange rides for patients without smartphones. This may improve time to discharge, patient satisfaction, and ED crowding.

## RESULTS

A total of 500 surveys were distributed; 480 (96%) subjects completed the survey and 20 subjects (4%) declined to participate after receiving the survey. The average age of the study subjects was 46.4 ± 18.7 years, and 291 (61%) were female. In contrast, the overall ED population during the study period had an average age of 40.0 ± 23.8 years, and 16,553 (49%) were female. Further demographics, including race, education, income, and smartphone/internet usage are displayed in table 1.

Of the 33,871 ED visits during the study period, 22,833 (67%) were discharged home. The most frequent mode of transportation to the ED was by ambulance (n = 127, 27%) followed by being dropped off by a family member or friend (n = 119, 25%) (Table 2).

Although 408 (85%) were aware of ridesharing services, only eight (2%) came to the ED by this manner, but 22 (5%) planned to use such a service post-discharge. There were 377 (79%) who indicated they possessed a smartphone, and 220 (46%) had previously used these ridesharing services. The highest number of subjects planned to get home with a family member or friend (n = 167, 35%), which was also the most preferred method (n = 141, 29%) (Table 2). Regarding the awareness of ridesharing services, we found significant age, education, and income differences from univariate analysis (Table 3). For prior use of ridesharing services, significant differences were found for only age and education (Table 4).

Logistic regression analysis of ridesharing awareness and use also revealed differences by age, education, and income, but not gender or race (Table 5). In general, both awareness and use decreased with age (Odds ratio (OR) less than one) and increased with rising education and income levels (OR greater than one). The model for predictors of ridesharing awareness was statistically significant (P < 0.0001, χ^2^ = 58.48, df = 17, Nagelkerke R^2^ = 0.20, and area under the receiver operating characteristic (ROC) curve 0.77 (95% confidence interval (CI), 0.72 to 0.80). Awareness predictor variables reaching statistical significance were age (OR = 0.96, P < 0.0001) education level up to 8^th^ grade (OR = 0.33, P = 0.03), income less than $20,000 per year (OR = 0.52, P = 0.02), and income $50,000 – $100,000 per year (OR =3.7, P = 0.01). The regression model for predictors of ridesharing use was also significant (P < 0.0001, χ^2^=86.11, df = 17, Nagelkerke R^2^ = 0.22, and area under the ROC curve 0.73 (95% CI, 0.69 to 0.77). Ridesharing use predictor variables reaching significance were age (OR = 0.95, P < 0.0001), high school or general educational development (GED) education level (OR = 2.05, P = 0.03), and college (OR = 2.77, P = 0.002). Income greater than $100,000 per year approached significance (OR = 2.09, P = 0.06).

Half the subjects (n = 241, 50%) felt their medical insurance should pay for their transportation home, whereas 148 (31%) felt the ED staff should arrange and pay for it. The average estimated distance home was 16.4 ± 22.0 miles, with most being less than 10 miles from the ED. There were 589 (2.6%) ridesharing transports arranged by ED staff and paid for by the hospital during the study period for a total cost of $8,731. Average cost and distance per ride was $15.70 ± 14.10 and 9.1 ± 10.1 miles, respectively. The majority were hailed during the day (7 AM – 7 PM) (n = 339, 57.6%) and 250 (42.4%) were hailed overnight (7 PM – 7 AM).

## DISCUSSION

Transportation to and discharge home from the ED is an essential need, especially for elderly, disabled, and economically disadvantaged patients. It is estimated roughly four million individuals fail to receive medical care annually due to transportation barriers.^5^ Delays in the diagnosis and treatment of patients with chronic diseases often results in destabilization and progression of those diseases, ED crowding, excessive use of inpatient resources, and poor outcomes.^6^–^8^ According to a 2016 report of the United States Government Accountability Office, the cost of medical transportation for Medicare and Medicaid patients exceeded $2.7 billion, with the Medicaid segment rising significantly in the past decade.^9^ This does not include estimates on the cost of missed and/or unused clinic appointments and negative downstream health effects, which is estimated at $150 billion.^10^

For the next several decades, the number and percentage of older adults are expected to increase, particularly the “oldest-old” (those 85 years and older). This subgroup will number roughly seven million (2% of the population) in 2020 but will grow to 18 million (4.5%) by 2050.^11^ Coupled with this aging population and rising Medicaid enrollment, government spending on medical transportation will continue to increase. Thus, the need for more cost-efficient ways to provide transportation for patients has become an important issue in healthcare and consumer spending.

Prior to the ridesharing app era, patients discharged from the ED who had no family/friends/self to drive them had to rely on taxis or private medical shuttle companies. At present, ridesharing companies such as Lyft and Uber have begun to offer programs that address this need at a lower cost than traditional taxi services, which have been shown to be more expensive in all major cities except New York.^12^ According to the Lyft business website, 80% of patients prefer Lyft for transportation, with a cost reduction of 32%.^13^ The chief business officer of Lyft wrote that the company’s goal is to reduce the healthcare transportation gap by 50% by 2020.^10^ Of 30,000 Lyft riders surveyed, 29% reported they have used the ridesharing app for healthcare transportation, according to the company’s 2019 economic impact report.^14^ Lyft has recently partnered with nine health systems and 10 medical transportation firms to provide patients with more extensive transportation options.^15^

Uber, the other major ridesharing app provider, launched Uber Health in 2015 for medical transportation.^16^,^17^ Uber Health, so far available only in the U.S., allows ED staff to book rides for discharged patients who do not have a smartphone. It has been used by more than 100 health facilities, many of which pay for the service to avoid the downstream health and personnel costs of delayed discharges and missed appointments. One issue that has arisen from the use of ridesharing apps for post-discharge transportation is that elderly and/or disabled patients may have unique needs and preferences, such as help getting into and out of the vehicle or a slower ride. Uber has responded to this need with Uber Assist, in which drivers are specifically trained to assist seniors and those with disabilities.^18^ Lyft has partnered with CareMore to provide similar services, and preliminary survey data from this program showed decreased transportation wait time and cost and increased patient satisfaction.^18^,^19^ Other novel programs aimed at reducing the transportation burden of older and disabled patients discharged from the ED include GoGoGrandparent, ITNAmerica, and Liberty Mobility Now.^20^

Despite the obvious cost and access advantages to the use of ridesharing services for medical transportation, the authors of one study found the impact of these services on medical appointment attendance may be minimal, even when offered for free. In their prospective clinical trial, Chaiyachati and colleagues offered gratis Lyft rides to 786 patients to and from their clinic appointments. The rate of missed appointments was 36.5% compared to 36.7% for study participants not offered free transportation.^21^ More than half of patients contacted with offers of a free ridesharing service responded they weren’t interested. Some theories on this finding were that those most in need of ridesharing, such as elderly and/or low-income patients, were the least technology-savvy and unlikely to own smartphones. Skepticism of ridesharing services and concern over privacy issues were also cited.

The findings of our survey with regard to age, income, and education parallel other studies conducted in non-healthcare settings. According to the Pew Research Center, 33% of adults in the U. S. have never heard of ridesharing services, and only 15% have ever used them.^22^ From the same survey, ridesharing users tended to be younger and college-educated, with higher than average incomes. Vivoda et al. surveyed older Americans and found 74% reported no knowledge of ridesharing services, and only 1.7% had used them.^20^ Younger age, male gender, and higher education were all independently associated with greater knowledge of ridesharing services in their study.

In the past, Lyft and Uber required the use of a smartphone and/or internet to hail a ride. It is estimated only 42% of older adults own a smartphone, and only 64% use the internet.^23^ Further survey findings have shown those with higher educations and incomes are likely to have more disposable income to spend on smartphones and internet access.^24^ In our study we did not ascertain differences between gender and race with regard to ridesharing service awareness and use. However, the authors of previously published studies have highlighted gender differences. Men, particularly in older adulthood, have been shown to take more trips per day than women and to have more favorable attitudes toward technology than women.^25^–^27^ Women may be less inclined to use ridesharing services, as it involves taking a ride with a stranger in an environment perceived as less regulated than a taxi.^28^

The use of immediately-available ridesharing services for transportation to and from the ED has several benefits. The first is eliminating the need for arranging a ride, driving to the ED, negotiating traffic, and finding parking. These actions add additional stressors upon the patient and their accompanying family and friends. Patients often receive sedating medications during their ED stay, such as antihistamines, antiemetics, benzodiazepines, and opioids. Ridesharing services may mitigate the risk associated with patients driving themselves home, especially the elderly, and these services have been shown to decrease substance-impaired driving after socialization.^29^,^30^ ED crowding may be favorably affected, as patient discharges from the ED no longer rely on finding a ride, which can take hours based on the availability of acquaintances or public transportation schedules.

When ridesharing services are hailed by the ED staff for a patient, as in our ED, there is no longer a need for patient ownership of a smartphone. Reduction of pollution, traffic congestion, and fuel use are further benefits. The transportation sector is the largest source (29%) of greenhouse gas emissions in the U.S., leading to serious air pollution and negative health effects, with cars alone accounting for the largest share (41.6%).^31^ Furthermore, over three-quarters of Americans drive alone to work, while 9.0% use ridesharing services and 5.1% use public transit.^32^ In heavily congested and polluted cities, such as Beijing, China, ridesharing has been shown to improve greenhouse gas emissions and energy savings.^33^

## LIMITATIONS

There are some limitations to this study that must be acknowledged. It is a survey study that relied on voluntary responses from subjects, although a high percentage (96%) completed the survey. Surveys were not distributed during the hours of midnight to 5 AM, and some differences in subject response may have been missed, especially during a time period of limited public and private transportation options. Recall bias may have been a factor, especially in the elderly subgroup. Some subjects may have been in discomfort or upset at ED crowding conditions while taking the survey, which may have affected their responses. Regarding the survey instrument (Supplement 1), response options were not alphabetized, which may have led some subjects to preferentially choose the first one or two options. There was overlap of income range on the fifth question, and the last question may have been misinterpreted by those subjects without medical insurance. Our ED serves an urban geographic area serving a population of over two million, and this may not reflect other urban or rural settings with different racial and ethnic proportions.

Another limitation is that our study subjects differed from the overall ED population with regard to gender and age, which may affect the generalizability of our findings. The proportion of females responding to the survey was significantly different from the overall ED population (60% vs 49%, X^2^ = 17.9, P < 0.0001). One potential explanation for this gender difference is that females are more likely than males to complete surveys.^34^ The average age of the study population was higher than the overall ED population (46.4 ± 18.7 versus 40.0 ± 23.8 years, P < 0.0001, unpaired t-test), which likely reflects that patients less than 18 years of age were excluded. Finally, this study was only able to determine demographics, preferences, and rates of knowledge and/or use of ridesharing services; it did not assess underlying socioeconomic or medical reasons for any observed differences.

## CONCLUSION

Patients prefer to be driven home by a family member or friend after discharge from the ED. There is ample awareness of ridesharing services, but only 5% use these services to get home after discharge from the ED. Patients who are older, have less income, and have less education are less likely to be aware of or have previously used ridesharing services. ED staff may suggest or even contact a ridesharing service for patients at time of discharge to assist in their transportation home. The on-demand and expeditious nature of ridesharing services may have a positive impact on patient satisfaction and ED crowding. Further studies are needed to assess these variables as the prevalence and success of ridesharing services continues to grow.

## Supplementary Information



## Figures and Tables

**Table 1:** Demographics of respondents to a survey of transportation preferences post discharge from the emergency department.

Age (years)	46.4 ± 18.7
Gender	
Female	291 (60.6%)
Male	186 (38.8%)
Undisclosed	3 (0.6%)
Race	
White	214 (44.6%)
Black	91 (19.0%)
Hispanic	80 (16.7%)
Asian	48 (10%)
Other	40 (8.3%)
Prefer not to disclose	7 (1.4%)
Education	
College	207 (43.1%)
High school or GED	172 (35.8%)
Graduate	47 (9.8%)
Vocational school	25 (5.2%)
Up to Grade 8	18 (3.7%)
Prefer not to disclose	11 (2.4%)
Income	
Less than $20,000	191 (39.7%)
$20,001 to $50,000	98 (20.4%)
$50,001 to $100,000	69 (14.4%)
Prefer not to disclose	85 (17.8%)
greater than $100,000	37 (7.7%)
Internet	
Own smartphone	377 (78.5%)
Text messages per day	21.9 ± 35.7
Emails sent per day	8.3 ± 20.1
Aware of rideshare apps?	408 (85%)
Used rideshare apps?	220 (45.8%)

*GED,* general educational development.

**Table 2:** Transportation to the emergency department, and plans/preferences for discharge transportation.

How did you come to the emergency department?	
Ambulance	127 (26.5%)
Dropped off by family member or friend	119 (24.8%)
Your personal vehicle driven by someone else	101 (21.0%)
Your personal vehicle alone	93 (19.4%)
Walk	16 (3.3%)
Public transportation (Bus/Light Rail)	12 (2.5%)
App-based rideshare service (Uber/Lyft)	8 (1.7%)
Taxi	3 (0.6%)
Bike	1 (0.2%)
How do you plan to get home?	
Pick-up by family or friend	167 (34.8%)
Your personal vehicle driven by someone else	119 (24.8%)
Your personal vehicle alone	89 (18.6%)
Not sure yet	28 (5.8%)
App-based rideshare services (Uber/Lyft)	22 (4.6%)
Walk	17 (3.5%)
Public transportation (Bus/Light Rail)	15 (3.1%)
Ambulance transport	11 (2.3%)
Taxi	8 (1.7%)
Other	3 (0.6%)
Bike	1 (0.2%)
Ideally, what is your top preference of transportation home?	
Pick-up by family or friend	141 (29.3%)
Your personal vehicle alone	140 (29.1%)
Your personal vehicle driven by someone else	105 (21.9%)
App-based rideshare services (Uber/Lyft)	26 (5.4%)
Public transportation (Bus/Light Rail)	19 (4.0%)
Taxi	13 (2.7%)
Free, hospital-provided shuttle	10 (2.1%)
Ambulance transport	8 (1.7%)
Other	8 (1.7%)
Walk	7 (1.5%)
Bike	3 (0.6%)

**Table 3:** Patient awareness of ridesharing services.

	Aware	Not aware	P
Age (years)	44.8 ± 18.1	55.7 ± 19.2	< 0.0001*
Gender			
Female	253 (86.9%)	38 (13.1%)	
Male	153 (82.3%)	33 (17.7%)	0.2
Race			
Asian	36 (75.0%)	12 (25.0%)	
Black	80 (87.9%)	11 (12.1%)	
Hispanic	63 (78.7%)	17 (21.3%)	
White	186 (86.9%)	28 (13.1%)	
Other	37 (92.5%)	3 (7.5%)	0.06
Education			
Up to Grade 8	11 (61.1%)	7 (38.9%)	
High School or GED	144 (83.7%)	28 (16.3%)	
Vocational School	19 (76.0%)	6 (24.0%)	
College	183 (88.4%)	24 (11.6%)	
Graduate	43 (91.4%)	4 (8.6%)	0.009
Income/year			
Less than $20,000	155 (81.1%)	36 (18.9%)	
$20,001 to $50,000	86 (87.7%)	12 (12.3%)	
$50,001 to $100,000	65 (94.2%)	4 (5.8%)	
Greater than $100,000	34 (91.8%)	3 (8.2%)	0.03

* Student’s t-test; otherwise *X*^2^.

*GED,* general educational development.

**Table 4:** Prior use of ridesharing services by emergency department patients.

	Used before	Never used	P
Age (years)	40.2 ± 17.4	51.6 ± 18.2	< 0.0001*
Gender			
Female	140 (48.1%)	151 (51.9%)	
Male	79 (42.7%)	106 (57.3%)	0.3
Race			
Asian	27 (56.3%)	21 (43.7%)	
Black	42 (46.1%)	49 (53.9%)	
Hispanic	32 (40.0%)	48 (60.0%)	
White	93 (43.4%)	121 (56.6%)	
Other	22 (55.0%)	18 (45.0%)	0.2
Education			
Up to Grade 8	4 (22.2%)	14 (77.8%)	
High School or GED	72 (41.8%)	100 (58.2%)	
Vocational School	6 (24.0%)	19 (76.0%)	
College	102 (49.3%)	105 (50.7%)	
Graduate	32 (68.1%)	15 (31.9%)	0.0004
Income/year			
Less than $20,000	79 (41.3%)	112 (58.7%)	
$20,001 to $50,000	49 (50.0%)	49 (50.0%)	
$50,001 to $100,000	35 (50.7%)	34 (49.3%)	
Greater than $100,000	22 (59.4%)	15 (40.6%)	0.1

* Student’s t-test; otherwise *X*^2^.

GED, general educational development.

**Table 5:** Multiple logistic regression analysis of ridesharing service awareness and use.

	B	SE	Wald	OR	95% CI	P
Awareness						
Age	−0.04	0.01	23.5	0.96	0.94 to 0.97	<0.0001
Education						
Up to Grade 8	−1.1	0.53	4.28	0.33	0.11 to 0.94	0.03
Vocational school	−0.56	0.97	0.33	1.18	0.23 to 5.96	0.83
High School/GED	−0.23	0.84	0.07	1.92	0.48 to 7.72	0.35
College	0.11	0.85	0.01	2.85	0.70 to 11.52	0.13
Graduate school	0.51	1	0.25	4.03	0.75 to 21.55	0.1
Income/year						
< $20,000	−0.64	0.27	5.4	0.52	0.30 to 0.90	0.02
$20,001 – $50,000	0.29	0.45	0.4	1.34	0.54 to 3.28	0.15
$50,001 – $100,000	1.31	0.62	4.42	3.7	1.09 to 12.51	0.01
> $100,000	1.08	0.72	2.25	2.95	0.71 to 12.16	0.11
Constant	3.26	1.42	5.22			0.02
Use						
Age	−0.04	0.01	45.64	0.95	0.94 to 0.97	<0.0001
Education						
Up to Grade 8	−1.03	0.93	1.24	0.35	0.05 to 2.19	0.26
Vocational school	−1.02	0.87	1.38	0.35	0.06 to 1.97	0.23
High School or GED	0.72	0.34	4.32	2.05	1.04 to 4.06	0.03
College	1.02	0.34	9	2.77	1.42 to 5.40	0.002
Graduate school	1.8	0.44	16.81	6.09	2.56 to 14.46	<0.0001
Income/year						
< $20,000	0.01	0.26	0.001	1.01	0.59 to 1.69	0.97
$20,001 – $50,000	0.35	0.29	1.42	1.42	0.79 to 2.56	0.23
$50,001 – $100,000	0.38	0.32	1.39	1.47	0.77 to 2.78	0.23
> $100,000	0.73	0.4	3.4	2.09	0.95 to 4.59	0.06
Constant	2.12	1.17	3.25			0.07

*B,* coefficient; *CI,* confidence interval; *GED,* general educational development; *SE,* standard error; *OR,* odds ratio.

## References

[1] Centers for Disease Control and Prevention (2017). Emergency department visits.

[2] Chang AM, Cohen DJ, Lin A (2018). Hospital strategies for reducing emergency department crowding: a mixed-methods study. Ann Emerg Med.

[3] Patel H, Morduchowicz S, Mourad M (2017). Using a systematic framework of interventions to improve early discharges. Jt Comm J Qual Patient Saf.

[4] Hathaway I, Muro M (2017). Ridesharing hits hyper-growth.

[5] Syed ST, Gerber BS, Sharp LK (2013). Traveling towards disease: transportation barriers to health care access. J Community Health.

[6] Erskine NA, Waring ME, McManus DD (2018). Barriers to healthcare access and long-term survival after an acute coronary syndrome. J Gen Intern Med.

[7] Euceda G, Kong WT, Kapoor A (2018). The effects of a comprehensive care management program on readmission rates after acute exacerbation of COPD at a community-based academic hospital. Chronic Obstr Pulm Dis.

[8] Chan KE, Thadhani RI, Maddux FW (2014). Adherence barriers to chronic dialysis in the United States. J Am Soc Nephrol.

[9] Government Accountability Office (2016). Nonemergency medical transportation: updated Medicaid guidance could help states.

[10] Baga D (2018). Nobody’s health should suffer because they can’t find a ride.

[11] Ortman JM, Velkoff VA, Hogan H (2014). An aging nation: the older population in the United States.

[12] Ride Guru (2017). Uber vs. Lyft vs. taxi: cost analysis across the United States.

[13] Lyft Concierge (2018). https://www.lyftbusiness.com/healthcare.

[14] Cohen JK (2019). % of Lyft riders use the app for healthcare trips, survey finds.

[15] Lyft (2016). New solutions to keep seniors moving.

[16] Uber Health (2018). https://www.uberhealth.com/.

[17] Collier R (2018). Uber enters medicine but disrupting health care may prove difficult. CMAJ.

[18] Powers BW, Rinefort S, Jain SH (2016). Nonemergency medical transportation: delivering care in the era of Lyft and Uber. JAMA.

[19] Joszt L (2018). CareMore finds success using Lyft to transport Medicare beneficiaries to appointments.

[20] Vivoda JM, Harmon AC, Babulal GM (2018). E-hail (Rideshare) knowledge, use, reliance, and future expectations among older adults. Transp Res Part F Traffic Psychol Behav.

[21] Chaiyachati KH, Hubbard RA, Yeager A (2018). Association of rideshare-based transportation services and missed primary care appointments: a clinical trial. JAMA Intern Med.

[22] Smith A (2016). On-demand: ride-hailing apps.

[23] Anderson M, Perrin A (2017). Technology use among seniors.

[24] Elliot AJ, Mooney CJ, Douthit KZ (2014). Predictors of older adults’ technology use and its relationship to depressive symptoms and well-being. J Gerontol B Psychol Sci Soc Sci.

[25] Zhang Y, Zhang Y (2018). Exploring the relationship between ridesharing and public transit sse in the United States. Int J Environ Res Public Health.

[26] Cai Z, Fan X, Du J (2017). Gender and attitudes toward technology use: a meta-analysis. Computers & Education.

[27] Goswami A, Dutta S (2016). Gender differences in technology usage—a literature review. Open J Business Management.

[28] Rogers B (2017). The social costs of Uber. University of Chicago Law Review Dialogue.

[29] Dickerson AE, Molnar LJ, Bédard M (2019). Transportation and aging: an updated research agenda to advance safe mobility among older ddults transitioning from driving to non-driving. Gerontologist.

[30] Whitehill JM, Wilner M, Rataj S, Moreno MA (2018). College students’ use of transportation networking companies: An opportunity to decrease substance-impaired driving. J Am Coll Health.

[31] United States Environmental Protection Agency (2019). Sources of greenhouse gas emissions.

[32] Tomer A (2017). America’s commuting choices: 5 major takeaways from 2016 census data.

[33] Yu B, Ma Y, Xue M (2017). Environmental benefits from ridesharing: a case of Beijing. Appl Energy.

[34] Slauson-Blevins K, Johnson KM (2016). Doing gender, doing surveys? Women’s gatekeeping and men’s non-participation in multi-actor reproductive surveys. Sociol Inquiry.

