# Supplementary material for: Transportation Preferences of Patients Discharged from the Emergency Department in the Era of Ridesharing Apps
Source: West J Emerg Med. 2019 Jul 2;20(4):672–80. doi: 10.5811/westjem.2019.5.42762 (PMC6625690; doi:10.5811/westjem.2019.5.42762)
Supplement: Supplementary file 1 [file wjem-20-672-s001.docx]

**Figure 1.** The survey instrument.

**Transportation Preferences after Discharge from the Emergency Department**

This is an anonymous and voluntary survey

Hello,

You are being asked to participate in a research study by answering a series of questions to help determine transportation preferences in patients (or their parents in the case of minors) discharged home from the Emergency Department. Participation is voluntary and will in no way affect the care rendered during your emergency department visit, or any future visits you may have.

If you have any questions feel free to contact the principal investigator.

By completing the survey that follows you agree to participate in the survey.

**What is your age?**

_______ years (if under 90)

or

- 90 years or older

**What is your gender?**

- Female
- Male
- Other___________________
- Prefer not to disclose

**What is your race/ethnic background (circle one)**

- Asian
- Black
- Hispanic
- White
- Other___________________
- Prefer not to disclose

**What is your highest educational level?**

- Up to Grade 8
- High School or GED
- Vocational School
- College
- Graduate
- Prefer not to disclose

**What is your gross income?**

- Less than $20,000
- $20,000 to $50,000
- $50,000 to $100,000
- greater than $100,000
- Prefer not to disclose

**Do you own a smartphone?**

- Yes
- No

**On average, how many text messages do you send per day?** ________ or None

**On average, how many emails do you send per day?** _________ or None

**How did you come to the Emergency Department?** (circle one)

- Your personal vehicle alone
- Your personal vehicle driven by someone else
- Dropped off by Family Member or Friend
- Public Transportation (Bus/Light Rail)
- Walk
- Bike
- Taxi
- Ambulance
- App-based ride-share service (Uber/Lyft)
- Other________________________

**Are you aware of application based ride-sharing services (Uber/Lyft)?**

- Yes
- No

**If “Yes”–have you used these services before?**

- Yes
- No

**Can you guess how far your home is from our Emergency Department?** ____ miles

**How do you plan to get home?**

- Your personal vehicle alone
- Your personal vehicle driven by someone else
- Pick-up by Family or Friend
- Public transportation (Bus/Light Rail)
- Walk
- Bike
- Taxi
- App-based ride-share service (Uber/Lyft)
- Ambulance transport
- Not sure yet
- Other__________________

**Do you feel that the Emergency Department should arrange and pay for your transportation home?**

- Yes
- No

**Ideally, what is your top preference of transportation home? (choose one)**

- Your personal vehicle alone
- Your personal vehicle driven by someone else
- Pick-up by Family or Friend
- Public transportation (Bus/Light Rail)
- Walk
- Bike
- Taxi
- App-based ride-share service (Uber/Lyft)
- Ambulance transport
- Free hospital-provided shuttle
- Other__________________

**Do you feel that your medical insurance should pay for your transportation home?**

- Yes
- No

**Thank you for taking this anonymous survey!**
